# Legal Issues and ADHD: Risk Factors and Temperamental Characteristics

**DOI:** 10.1192/j.eurpsy.2025.1655

**Published:** 2025-08-26

**Authors:** F. Soricaro, G. Longo, M. Servasi, L. Orsolini, U. Volpe

**Affiliations:** 1 Unit of Clinical Psychiatry, Department of Clinical Neurosciences/DIMSC, Polytechnic University of Marche, Ancona, Italy

## Abstract

**Introduction:**

Adult Attention Deficit Hyperactivity Disorder (ADHD) has been recognized as a condition with profound psychosocial implications, including a higher likelihood of encountering legal problems.

**Objectives:**

To characterize temperamental characteristics and risk factor for legal problems in a group of ADHD patients.

**Methods:**

The study involved patients (>18 years) from the adult ADHD outpatient service of the Psychiatric Clinic of Ancona.ADHD was diagnosed using the Diagnostic Interview for ADHD in adults (DIVA5.0). The following rating scale were administered: Temperament Evaluation in Memphis, Pisa and San Diego(TEMPS-M), Coping Orientation to the Problems Experiences-new Italian version (COPE-NVI), Temperament and Character Inventory-Revised (TCI-R).

**Results:**

76% (n=170) of screened patients were diagnosed with ADHD in adulthood. Among them, 10.2% (n=17) reported legal problems. Men with ADHD were more likely to have legal issues (χ²=7.851; p=0.005). These patients often reported school failures(χ²=3.033; p=0.082) and being tutored by a support teacher(χ²=15.165; p<0.001). Legal issues were more common in those with a childhood diagnosis of ADHD (χ²=4.880; p=0.027) and in those who were admitted to a psychiatric ward (χ²=7.205; p=0.007).Additionally, those who smoked (χ²=6.695; p=0.035) or used substances (χ²=16.399; p<0.001) had more legal issues. Higher use of cannabinoids (χ²=14.311; p<0.001) and stimulants (χ²=12.445; p<0.001) in those who had legal problems are also reported. Generally, those with legal problems have lower schooling (p=0.005), more high school failures (p=0.047), smoke more cigarettes per day (p=0. 008) and have higher scores in the problem-solving subscale of the COPE-NVI (p=0.024), the ambitious subscale of the TCI-R (p=0.058), and the self-transcendence subscale of the TCI-R (p=0.078) and lower mean score in the self-acceptance subscale of the TCI-R (p=0.054). Logistic regression analysis showed to ascertain the effects of all TCI-R subscales, on the likelihood of developing legal problems. The logistic regression model was statistically significant, ▫▫2(1)=4.020, p=0.045. The model explained 14.6% (Nagelkerke R2) of the variance in patients with ADHD who have legal problems and correctly classified 91.9% of cases.Having legal problems was significantly predicted by TCI-R spiritual acceptance subscale (exp(B)=1.150, p=0.045).

**Conclusions:**

The study highlight the significant psychosocial burden of ADHD in adulthood, particularly the increased likelihood of legal problems.Key risk factors include lower educational attainment, substance use, and specific temperamental traits, such as elevated problem-solving and self-transcendence tendencies, alongside lower self-acceptance.

**Disclosure of Interest:**

None Declared

